# First report of a severe nasopulmonary acariasis caused by *Orthohalarachne diminuata* Doetschman, 1944 (Acari: Halarachnidae) in a captive South American sea lion (*Otaria flavescens* Shaw, 1800)

**DOI:** 10.1016/j.ijppaw.2022.10.005

**Published:** 2022-11-02

**Authors:** David Ebmer, Edwin Kniha, Verena Strauss, Anna Kübber-Heiss, Lukas Komornik, Folko Balfanz, Stephan Hering-Hagenbeck, Julia Walochnik, Ulrich Gärtner, Heinrich Prosl, Anja Taubert, Thomas Voracek, Carlos Hermosilla

**Affiliations:** a Veterinary Clinic Vienna Zoo, Seckendorff-Gudent-Weg 6, 1130, Vienna, Austria; b Institute of Parasitology, Biomedical Research Center Seltersberg, Justus Liebig University Giessen, Schubertstr. 81, 35392, Giessen, Germany; c Institute of Specific Prophylaxis and Tropical Medicine, Center for Pathophysiology, Infectiology and Immunology, Medical University of Vienna, Kinderspitalg. 15, 1090, Vienna, Austria; d Research Institute of Wildlife Ecology, Department for Interdisciplinary Life Sciences, Vetmeduni Vienna, Savoyenstr. 1, 1160, Vienna, Austria; e Vienna Zoo, Maxingstr. 13b, 1130, Vienna, Austria; f Institute of Anatomy and Cell Biology, Justus Liebig University Giessen, Aulweg 123, 35385, Giessen, Germany

**Keywords:** Marine mammals, Halarachnids, Parasites, Respiratory mites, Zoological garden

## Abstract

Obligatory endoparasitic mites of the genera *Halarachne* Allman, 1847 and *Orthohalarachne* Newell, 1947 (Acari: Halarachnidae) parasitize different segments of the respiratory tract of marine mammals, including pinnipeds and sea otters, and infestations can cause asymptomatic to serious respiratory diseases. However, knowledge on biology, pathogenic potential and occurrence of halarachnid mites infesting pinnipeds, especially in captivity, is scarce. A two-year-old South American sea lion (*Otaria flavescens* Shaw, 1800) male, born and held at the Vienna Zoo, was anesthesized for routine pre-transport examinations, including computed tomography, bronchoalveolar lavage, and blood sampling. During the final phase of general anesthesia, the individual abruptly became apneic and died despite all attempts at resuscitation. At necropsy, 45 highly motile whitish millimeter-sized structures were macroscopically detected in the trachea, *bifurcatio tracheae* and main bronchi and were identified as adult stages of *Orthohalarachne diminuata* Doetschman, 1944 following morphological descriptions. After trepanation of the nasal cavity and *sinus paranasalis*, a total of 407 larval and 3 nymphal specimens distributed in clusters were detected. Macroscopically, sinus mucosa showed hyperemia and multiple petechial hemorrhages. Histopathological analyses of paranasal sinuses revealed mite cross-sections surrounded by sanioserous exudate and epithelial exfoliation. For the first time, *O. diminuata* was molecularly characterized and phylogenetically analyzed based on its 16S rDNA. Our study constitutes the first record of a severe *O. diminuata* infestation in captive *O. flavescens* and one of the few host-parasite records in general. We present clinical data and pathological results, the first scanning electron microscopic images of a *O. diminuata* larval stage and discuss the etiology of this autochthonous infestation, possible transmission pathways and detrimental effects. Further studies on biology and pathogenic effects of halarachnid mites, as well as on the development of non-invasive sampling techniques are essentially required for a better understanding of (ortho-)halarachnosis in pinnipeds held in zoological gardens.

## Highlights

• First record of *Orthohalarachne diminuata* in a captive South American sea lion.
• One of the few host-parasite records.
• Autochthonous infestation, since infested animal was born in captivity.
• First scanning electron microscopic images of a *O. diminuata* larva.
• First molecular characterization and phylogenetic analysis of *O. diminuata*.

## Graphical abstract

**Figure:**
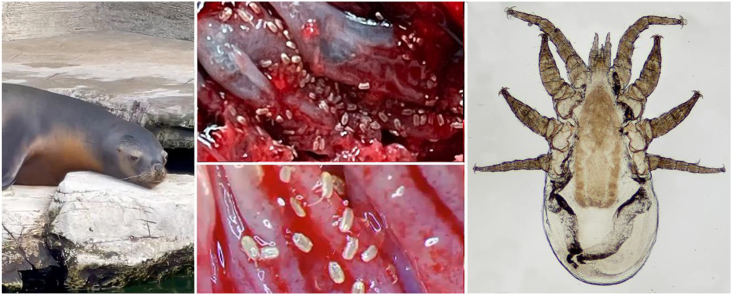

## 1 Introduction

Mesostigmatid mites of the genera *Halarachne* Allman, 1847 and *Orthohalarachne* Newell, 1947 belonging to the family Halarachnidae Oudemans, 1906 constitute obligatory endoparasites infesting the respiratory tract of different semiaquatic marine mammals. Thereby, representatives of the genus *Halarachne* parasitize pinnipeds of the family Phocidae Gray, 1821 (earless seals) and sea otters (*Enhydra lutris* Linnaeus, 1758), while the genus *Orthohalarachne* occurs in pinnipeds of the order Otariidae Gray, 1825 (eared seals) and Odobenidae Allen, 1880 (walruses) (Kim et al., 1980; Rolbiecki et al., 2018; Seguel et al., 2018a; Reckendorf et al., 2019; Pesapane et al., 2021). Within the genus *Orthohalarachne*, two species (with various synonyms) have been described: *O. attenuata* Banks, 1910 infesting nasopharyngeal mucosa, and *O. diminuata* Doetschman, 1944 parasitizing in parts of the upper and the lower respiratory tract (Kim et al., 1980; Rolbiecki et al., 2018). In free-ranging otariids, high prevalences of *Orthohalarachne* spp. have been reported, e.g. 48.6–100% in South American fur seals (*Arctocephalus australis* Zimmermann, 1783) (Páez, 2007; Gastal et al., 2016; Seguel et al., 2018b), 74.1–85% in California sea lions (*Zalophus californianus* Lesson, 1828) (Kuzmina et al., 2018; Pesapane et al., 2021), and 73.3–100% in Northern fur seals (*Callorhinus ursinus* Linnaeus, 1758) (Dunlap et al., 1976; Kim et al., 1980; Kuzmina et al., 2021; Pesapane et al., 2021). Co-infestations of both *Orthohalarachne* species are thereby regularly recorded (Kim et al., 1980; Gastal et al., 2016).

Adult *O. diminuata* mites are mainly found in the trachea, bronchi and bronchioles, whereas larval and nymphal stages are mostly detected in nasal cavities and paranasal sinuses (Kim et al., 1980). Highly motile hexapod larvae represent epidemiologically relevant transmission stages in the monoxenous life cycle and are transmitted via direct nose-to-nose contact between hosts or via mucus droplets containing mites, which are expectorated during intensive sneezing and coughing (Dahme and Popp, 1963; Furman and Smith, 1973). It has been proposed that exogenous CO_2_ atmosphere gradients can function as attractant for expectorated mites, which are not in immediate proximity of the nostrils of new host individuals (Furman and Smith, 1973). Under zoo-held conditions, indirect transmission via mechanical vectors (e.g. hands of zookeepers during animal training) or environmental contamination with larval mites has also been suggested as alternative possible pathways (Fravel and Procter, 2016; Pesapane et al., 2018). Larval stages are followed by two nymphal stages (i.e. octopod proto- and deutonymphs), which morphologically show reduced tarsal claws, consequently are less motile and only have a short duration (Furman and Smith, 1973). For completion of the life cycle, octopod nymphal or freshly molted adult stages are migrating from nasal cavities to the lower respiratory tract for mating purposes (Kim et al., 1980). Using their sharp chelicerae and pedipalpes, mites can scratch and injure epithelial layers of respiratory mucosa (Dahme and Popp, 1963).

Infestations with halarachnid mites can cause a range of asymptomatic up to serious respiratory diseases in infested pinnipeds and sea otters (Dahme and Popp, 1963; Dunlap et al., 1976; Kim et al., 1980; Geraci and Aubin, 1987; Shockling Dent et al., 2019). Damage of respiratory mucosa, respiratory obstructions and even fatal pulmonary pathologies including bronchitis and secondary alveolar emphysema have been associated with *O. diminuata* infestations in sea lions and fur seals (Dahme and Popp, 1963; Kim et al., 1980; Seguel et al., 2018a). Respiratory mucosa damages caused by *O. diminuata* could also promote secondary bacterial or fungal infections of the lungs (Seguel et al., 2018a). However, the exact pathogenicity of nasopharyngeal/nasopulmonary acariasis in free-ranging and zoo-housed pinnipeds and even the potential vector function of halarachnid mites is still vastly understudied (Kim et al., 1980; Pesapane et al., 2022).

In the present study, we report a severe *O. diminuata* infestation in a captive 2-year-old South American sea lion (*Otaria flavescens* Shaw, 1800) male, held at Vienna Zoo, that suddenly died in the final phase of general anesthesia for routine pre-transport examinations. Thereby, the study presents clinical and pathological data as well as morphological mite identifications and, for first time, molecular characterization and phylogenetic analysis of *O. diminuata*.

## 2 Materials & methods

### 2.1 Animal history

The 2-year-old South American sea lion male was born in June 2019 at Vienna Zoo, Austria. The parents of this individual were also born in captivity: The dam was born in July 2013 and transported to Vienna Zoo in May 2014, while the sire was born at Vienna Zoo in July 2002 and euthanized in March 2021 due to multicentric lymphoma. At time of study, the group of South American sea lions held at Vienna Zoo consisted of seven individuals (one harem leading adult male, three females, one subadult male and two juvenile males). The animal had a body weight of 115 kg. The *Otaria flavescens* enclosure is located next to the exhibits of Humboldt penguins (*Spheniscus humboldti* Meyen, 1834), Northern rockhopper penguins (*Eudyptes moseleyi* Mathews and Iredale, 1921) and king penguins (*Aptenodytes patagonicus* Miller, 1778). At time of study, *O. flavescens* was the only pinniped species housed at the zoo.

### 2.2 Ante mortem examinations

In November 2021, various routine diagnostics of the subadult male were carried out for an upcoming transport of this animal within the European Endangered Species Programme (EEP): blood sampling, computed tomography (CT) with a focus on the pulmonary system, and video endoscopy-assisted bronchoalveolar lavage (BAL) mainly for evaluation of *Mycobacterium pinnipedii*-infection status. Therefore, general anesthesia using atropine (1 mg in toto), midazolame (0.13 mg/kg), medetomidine (0.014 mg/kg) and butorphanole (0.05 mg/kg) was applied via injection into the femoral musculature. The animal was intubated and received a permanent oxygen feed. A peripheral vein catheter was placed and anesthesia was maintained using propofol (10 mg/ml titrated to effect) and isoflurane. The animal was transported to the zoo's veterinary clinic (5–10 min transit time). All planned diagnostic examinations were carried out on schedule and the animal was returned to the indoor enclosure, where anesthetic antagonists (atipamezole and yohimbin) were administered. In the final stage of anesthesia, the animal suddenly became apneic and died despite all attempts at resuscitation (cardiac massage and intracardiac administration of atipamezole and epinephrin). Prior to these pre-transport-examinations, the patient was not observed to show any clinical symptoms and had no relevant medical history.

### 2.3 Post mortem examinations

Immediately after death, the animal was transported to the Pathology Unit of the Research Institute of Wildlife Ecology (Vetmeduni Vienna), Vienna, Austria and necropsy was performed. Macroscopically visible mites in the respiratory tract were documented, i. e. filmed and photographed, extracted and preserved in 80% ethanol for further examinations. Beside gross pathology, samples from different tissues and sterile swaps were taken for histopathological and bacteriological analysis, respectively.

### 2.4 Morphological identification and light microscopy of halarachnid mites

Preserved nasopulmonary mites were mounted in Hoyer's medium, examined and documented after 48 h using an Olympus BX50 light microscope (Olympus, Japan) equipped with a digital camera (Olympus EP50, Olympus, Japan). Morphological identification followed established diagnostic descriptions (Doetschman, 1944; Gretillat, 1959; Popp, 1961; Domrow, 1974).

### 2.5 Scanning electron microscopy (SEM) analysis

A larval *O. diminuata* specimen was transferred onto a coverslip pre-coated with poly-L-lysine (0.01%; 10 mm/diameter; Thermo Fischer Scientific, Braunschweig, Germany). The sample was fixed in 2.5% glutaraldehyde (Merck, Darmstadt, Germany) and post-fixed with 1% osmium tetroxide (Merck, Darmstadt, Germany), rinsed in distilled water, dried, CO_2_-treated to the critical point and sputter coated with gold. The sample was analyzed by use of a scanning electron microscope (Philips XL30, Eindhoven, The Netherlands) at the Institute of Anatomy and Cell Biology, Justus Liebig University Giessen, Giessen, Germany.

### 2.6 Molecular characterization and phylogenetic analysis

DNA was isolated from individual specimens with a QIAamp® DNA Mini Kit 250 (Qiagen, Hilden, Germany) with a final elution volume of 100 μL.

For molecular characterization, initial PCR amplification of a fragment of the 16S rRNA gene was attempted by using the primer combination 16S + 1/16S-1. Since this combination did not result in any amplificates, the combinations 16S + 1/16S-2 and 16S + 2/16S-1 were tested to amplify overlapping fragments following the protocol of Black and Piesman (1994). Of these, only the second primer combination lead to gene fragment amplification (16S + 2/16S-1). Therefore, a new specific *Orthohalarachne* forward primer for the first fragment, 16S + 1_orthohala (5′-GCTGTGGGATCATTTACCG-3′), was designed based on aligned Halarachnidae sequences available in GenBank (see Additional file 1: Fig. S1). Oligo Calc was used to calculate GC-contents, melting temperatures, optimal primer lengths and to exclude self-complementarity (http://biotools.nubic.northwestern.edu/OligoCalc.html). Primer combinations 16S + 1_orthohala/16S-1 as well as 16S + 1_orthohala/16S-2 gave positive PCR results when using the following PCR conditions: 95 °C for 15 min, followed by 38 cycles of 95 °C for 1 min (denaturation), 52 °C for 1 min (annealing) and 72 °C for 1 min (elongation), followed by a final extension at 72 °C for 10 min.

Amplification by PCR was performed in 10 x Reaction Buffer B with 2.5 mM MgCl_2_, 1.6 mM dNTPs, 1 μM primers, 1.25 units DNA polymerase and 5 μL DNA. Sterile H_2_O was added to a final volume of 50 μL.

All PCR amplifications were run on an Eppendorf Mastercycler (Eppendorf AG, Hamburg, Germany). Bands were analyzed with a Gel DocTM XR + Imager (Bio-Rad Laboratories, Inc., California, U.S.A.) and respective bands cut out from the gel and purified with an IllustraTM GFXTM PCR DNA and Gel Purification Kit (GE Healthcare, Buckinghamshire, UK). Sanger sequencing was performed with a Thermo Fisher Scientific SeqStudio (Thermo Fisher Scientific, Massachusetts, USA). Sequences were obtained from both strands and a consensus sequence was generated in GenDoc 2.7.0.

The obtained sequences were compared to available sequences in the GenBank database using the Basic Local Alignment Search Tool (BLAST) (https://blast.ncbi.nlm.nih.gov/Blast.cgi).

Available sequences for comparison were downloaded from GenBank and aligned with the obtained sequences using ClustalX 2.1 for multiple alignment and GeneDoc 2.7.0. For manual editing and data analysis. For phylogenetic inference pairwise distances and maximum likelihood (ML) analysis were calculated in MEGA X (Kumar et al., 2018). Based on best fit evolutionary model selection, Tamura's-3-parameter + G model was applied with 1000 bootstrap support.

## 3 Results

### 3.1 Pathological findings

**Fig. 1:**
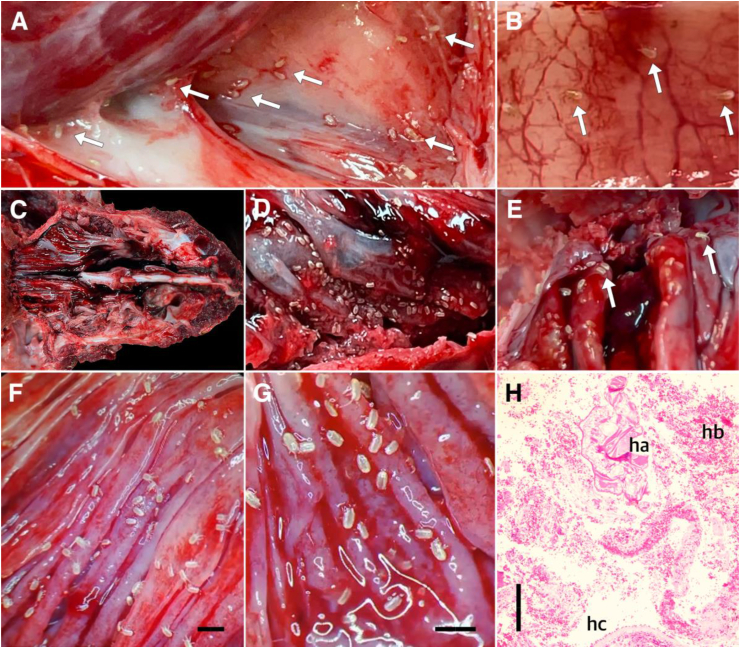
**Severe *Orthohalarachne diminuata* infestation in a 2-year-old South American sea lion.** (A) *Bifurcatio tracheae* reveals various adult mites migrating out of bronchial system. (B) Hyperemic tracheal vessels and adult mites. (C) Trepanation of nasal cavity and *sinus paranasalis*. (D, E) Multiple clusters of larval mites infesting turbinate mucosa. (F, G) Larval mites burying their pedipalpes and legs into the turbinate mucosa and causing multiple petechial hemorrhages and hyperemia of mucosa. (H) Histological cross-section of turbinate mucosa, showing larval mite (ha), surrounded by saniserous exsudate (hb), and epithelial exfoliation (hc). Legend: White arrows = *O. diminuata*. Scale bars: (F, G) 2 mm, (H) 200 μm.

The dissection of the respiratory tract, including opening of the trachea, *bifurcatio tracheae* and anterior parts of the bronchial tree, revealed the presence of multiple whitish and millimeter-sized specimens migrating through different parts of lower airway anatomy (Fig. 1A and B). These arthropod-like dot-shaped moving objects were very active and crawled dynamically through the respiratory tract, while further structures steadily appeared from posterior non-opened parts of the airways. The tracheal mucosa exhibited moderate mucus accumulations and showed moderate, partially considerable, hyperemia (Fig. 1B). The lungs were considerably hyperemic, in some spots dystelectatic, while hemorrhages were occasionally found in bronchi.

Following initial findings of supposed endoparasites in the lower respiratory tract, a parasitologist was consulted. The whitish structures were interpreted as nasopulmonary mites and the opening of the nasal cavity and sinus paranasalis was disposed (Fig. 1C). Immediately after trepanation, we identified massive mite clusters evenly distributed throughout the entire turbinate mucosa (Fig. 1D–G). Macroscopically, the turbinate mucosa was hyperemic and showed mucus accumulation and multiple petechial hemorrhages (Fig. 1F and G).

Histological analysis of the turbinate mucosa revealed various cross-sections of mites being surrounded by saniserous exsudate and epithelial exfoliation (Fig. 1H). By histopathological examination of the lungs, a focal low-grade *peribronchitis follicularis* was diagnosed. Bacteriological analysis of tracheal and lung swabs revealed presence of *Streptococcus dysgalactiae equisimilis*.

### 3.2 Parasitological findings and morphological identification of nasopulmonary mites

**Fig. 2:**
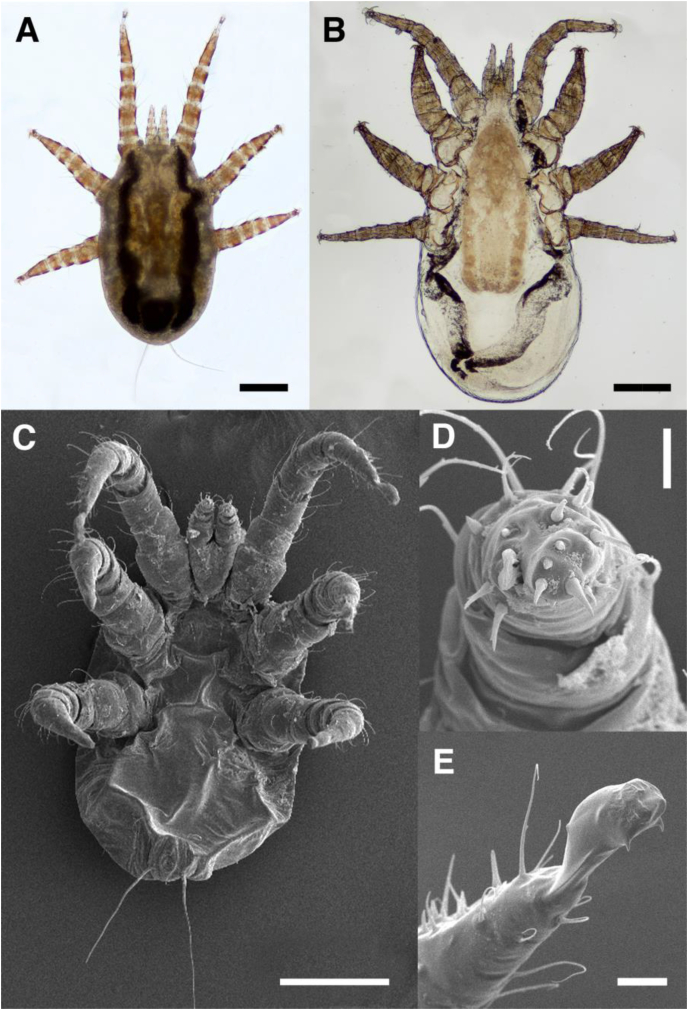
**Morphological identification of *Orthohalarachne diminuata***. Light microscopy of (A) hexapod larval stage and (B) adult female. (C, D, E) Scanning electron microscope (SEM) images of (C) a larval stage, revealing (D) the anterior end of a pedipalp and (E) claw of first leg, showing hair-like sensillae. Scale bars: (A, B, C) 200 μm, (D) 10 μm, (E) 20 μm.

Endoparasite specimens were morphologically identified as *Orthohalarachne diminuata* (Fig. 2). Overall, 455 *O. diminuata* mites were extracted from the upper and lower respiratory tract and all stages (i.e. 407 larval, 3 nymphal and 45 adult mites) were detected. Thereby, 45 adult mites (41 females, 4 males) were collected from the lower respiratory tract, including the trachea, *bifurcatio tracheae* and main bronchi (Fig. 1A and B). Moreover, 407 larvae and 3 nymphs were extracted exclusively from the upper respiratory tract, colonizing the mucosa of the nasal cavity and *sinus paranasalis* and forming distinct clusters (Fig. 1D). SEM-images of a larval stage revealed detailed views of general morphology (Fig. 2C), pedipalps (Fig. 2D) and claws of first leg (Fig. 2E).

### 3.3 Retrospective evaluation of ante mortem examinations

**Fig. 3:**
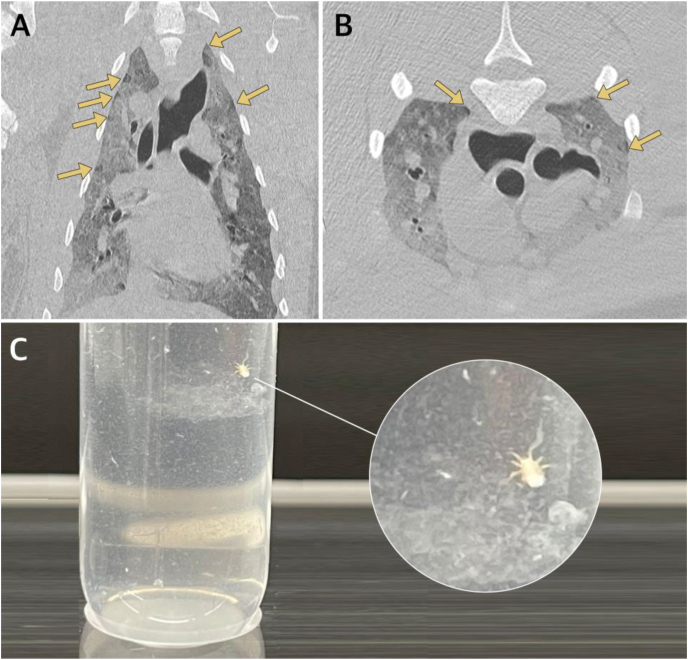
**Retrospective evaluation of ante mortem examinations.** (A) Dorsal and (B) cranial view of computed tomography (CT) of lung field, revealing marginal emphysematous bullae potentially associated with *O. diminuata* infestations (arrows). (C) Macroscopically visible mite and mucous in bronchoalveolar lavage (BAL) fluid.

By retrospective evaluation of previous CT-images, multiple marginal and almost round emphysematous bullae-like structures lacking radio-opacity were noticed (Fig. 3A and B). There were no signs of free gas in the thoracic cavity. Beside minor ventral atelectasis and the previously mentioned emphysematous bullae-like structures, the lung parenchyma showed no pathologies. Macroscopic exploration of 40 ml BAL-fluid revealed the occurrence of three mites, which were identified by light microscopy as two adult females and one hexapod larva (Fig. 3C).

### 3.4 Molecular characterization and phylogenetic analysis

To further characterize *O. diminuata*, 16S rDNA sequences of four larvae as well as four adults were obtained using the primer combination 16S + 1_orthohala/16S-1. All eight analyzed specimens resulted in identical sequences with a length of 424 bp including primers and 383 bp without primers. Two sequences of larval and adult stages each were submitted to GenBank (accession numbers OP087659.1–OP087662.1). The obtained sequences were queried against available sequences in GenBank by BLAST. Since no *O. diminuata* sequences were available in GenBank, closest hits were obtained to *O. attenuata* showing 86.48% (MZ435794.1) to 86.79% (MZ435797.1) identity with 100% query cover.

For first and deeper molecular insights, seven sequences were included into pairwise distance analysis resulting in a 404 bp alignment including gaps. For comparison, available sequences of four Halarachnidae species (*O. diminuata*, *O. attenuata*, *Halarachne halichoeri* Allman, 1847; *H. miroungae* Ferris, 1925)*, Dermanyssus gallinae* De Geer 1778, *Ixodes ricinus* Linnaeus, 1758 and *Dermacentor reticulatus* Fabricius, 1794 were included in the analysis. Pairwise Tamura-3-parameter distances were calculated without gaps taking 369 sites into account. Pairwise distances between *O. diminuata* and other included species ranged from 12.5% to 34.7% and were lowest compared to *O. attenuata* and highest compared to *D. reticulatus* (Table 1).

**Table 1:** Pairwise Tamura-3-parameter distances (%) based on 16S rDNA sequences.

	Species analyzed	1	2	3	4	5	6	7
1	OP087659.1 *Orthohalarachne diminuata*	–						
2	MZ435793.1 *Orthohalarachne attenuata*	12.5	–					
3	MZ435788.1 *Halarachne halichoeri*	21.4	25.5	–				
4	MZ435791.1 *Halarachne miroungae*	20.6	24.4	4.1	–			
5	LC029796.1 *Dermanyssus gallinae*	24.7	26.6	22.0	23.0	–		
6	MH645521.1 *Ixodes ricinus*	30.9	32.0	31.2	32.8	26.8	–	
7	MH645514.1 *Dermacentor reticulatus*	34.7	35.8	32.0	33.3	29.8	19.8	–

**Fig. 4:**
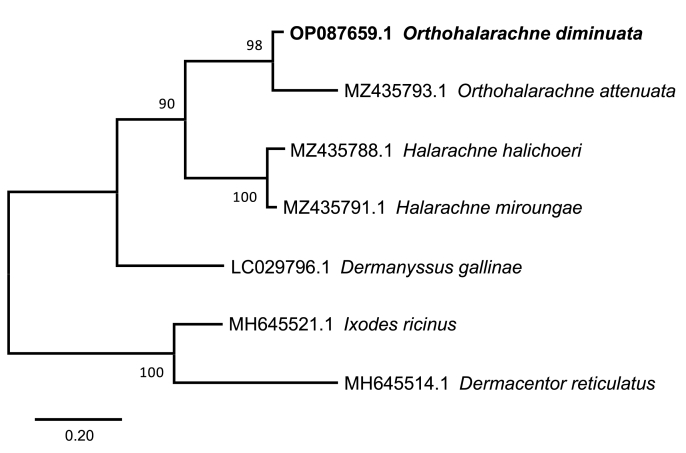
**ML tree based on 16S rDNA sequences.***I. ricinus* and *D. reticulatus* were used as outgroups. Bootstrap values of >70% are shown.

Despite limited availability of reference sequences, *Orthohalarachne* monophyly was tested by phylogenetic analysis, whereby *I. ricinus* and *D. reticulatus* were used as outgroups. *Orthohalarachne diminuata* and *O. attenuata* formed a clade, and confirming these two as sister species. Also, *H. halichoeri* and *H. miroungae* were revealed as sister species forming a second clade. These two clades displayed a monophyletic Halarachnidae group (Fig. 4).

## 4 Discussion

In the present study, we report a severe *Orthohalarachne diminuata* infestation in a zoo-housed subadult South American sea lion. While there are some records of *O. attenuata* infestations in free-ranging and captive *Otaria flavescens* (Finnegan, 1934; Kutzer and Burtscher, 1983; Morgades et al., 2006; Gomez-Puerta and Gonzales-Viera, 2015; Seguel et al., 2018b), to the best of our knowledge, only one record of *O. diminuata* mites parasitizing South American sea lions was published before (Calderón-Mayo, 2015). Accordingly, the current case constitutes one of the few host-parasite records and the first evidence of a severe *O. diminuata*-infestation in a captive South American sea lion, so far.

In general, reports on halarachnid mite infestations in captive pinnipeds are rare and most of these reports concern wild-caught, originally free-ranging, animals, e.g. *O. attenuata* in Pacific walruses (*Odobenus rosmarus divergens* Illiger, 1815) originating from St. Lawrence Island, Alaska (Fravel and Procter, 2016), *O. attenuata* (as *Halarachne rosmari* Oudemans, 1916) in a walrus (*O. rosmarus* Linnaeus, 1758) originating from Franz Joseph Land (Oudemans, 1916; Weidner, 1954), *O. diminuata* (as *O. chabaudi* Gretillat, 1959) in Antarctic fur seals (*Arctocephalus gazella* Peters, 1875) originating from Amsterdam Islands, Southern Indian Ocean (Gretillat, 1959) and *H. halichoeri* (as *H. taita* Eichler, 1958) in a Southern elephant seal (*Mirounga leonina* Linnaeus, 1758) originating from South Georgia (Eichler, 1958). However, in the current case, the animal was born in the zoo in 2019 and thereafter never left the Vienna Zoo, evidencing that this infestation was autochthonous and acquired under zoo conditions. Of note, the parent animals of the infested sea lion were also born in captivity and – within the last years - *O. flavescens* imports at the Vienna Zoo exclusively comprised captive-born individuals being transported within the framework of the European Endangered Species Programme (EEP). In 2014, a juvenile female (subsequently the dam of the infested animal) was imported, followed by a 3-year old male in 2017 and a 2-year old female in 2018, all originating from different German zoos. By retrospective screening of all *O. flavescens* laboratory data of BAL-cytology and internal tracheal-/bronchial-endoscopy-based records (conducted for routine pre-transport examinations and *Mycobacterium pinnipedii*-diagnostics) carried out at Vienna Zoo between 2002 and 2021, no halarachnid mite findings were recorded. Furthermore, no halarachnid mite infestations were listed in any animal transport certificates from other zoological facilities. Consequently, the actual infestation status of the remaining animals of the group is unclear and needs to be thoroughly monitored in follow-up studies in the near future.

Almost 40 years ago, Kutzer and Burtscher (1983) reported on *O. attenuata* infestations in two approximately one-year old South American sea lions held at the Vienna Zoo. The animals were probably wild-caughts and died a few weeks after their arrival at the zoo. During necropsy, mites were detected as incidental findings in caudal parts of the *pars respiratoria* of the *cavum nasi* and in the region of the *choanae* being associated with colliquative necrosis, plasma exudation and minor hemorrhages in the infestation area (Kutzer and Burtscher, 1983). In the current case, exclusively larval and nymphal stages of *O. diminuata* were detected in the sinus system after trepanation. Moreover, *O. attenuata* specimens were not found, thereby excluding a co-infestation. Unfortunately, it is impossible to reconstruct the relationship of these infested animals to the ancestors of the present zoo group members. Records in the historical archive of the Vienna Zoo showed that South American sea lions have been held since 1937 with interceptions during World War 2, and again from 1952 on. The report of Kutzer and Burtscher (1983) constitutes a textbook example of the unnoticed introduction of an asymptomatic and underdiagnosed endoparasitosis into zoological gardens, which was only revealed by necropsy, as it is also known for other symptom-less endoparasitoses of captive pinnipeds (Klotz et al., 2018). It is thereby plausible, that the introduction and establishment of *O. diminuata* within the zoo-held population of *O. flavescens* happened as follows: being initially introduced via wild-caught animals autochthonously infested within their natural habitats, asymptomatic or minor-symptomatic infested animals in good constitution might not have been recognized thereby allowing parasite transmission and spread. Consequently, new individuals developed patent infestations and the life cycle established over time under captivity-conditions. During necropsies, low parasite burdens were potentially overlooked in the past, since opening of nasopharyngeal cavities is not part of common necropsy protocols. This is aggravated by the fact that larval populations of *Orthohalarachne* spp.*,* in case of *O. diminuata* exclusively located in the upper respiratory tract, can represent up to 99% of the total mite burden (Kim et al., 1980). Additionally, comparably few whitish millimeter-sized adults might be pulled back into the bronchioles and are macroscopically inconspicuous. In this context, a potentially low mite burden might not have been noticed during the necropsy of the 18-year-old sire of the infested animal, which was euthanized in March 2021, i. e. approximately 8 months before the current case was diagnosed. Pathological results revealed multicentric lymphoma. However, neither mite infestations nor other parasite infections were recorded. Thus, the current case, emphasizes the importance of accurate ante and post mortem examinations and simultaneously raises awareness to uncommon endo- and ectoparasitoses in captive animals. Thereby, the establishment of species-specific adaptations of wildlife/zoo animal necropsy protocols, e.g. mandatory trepanations and openings of nasal turbinates in pinnipeds, constitute important measures to also explore uncommon infection and/or infestation sites of neglected parasites in exotic wildlife species and zoo-housed animals.

In addition to the proposed main transmission pathways in free-ranging animals, e. g. via direct nose-to-nose contact or mite-containing expectorated mucus (Dahme and Popp, 1963; Furman and Smith, 1973), under zoo-held conditions, spreading of *O. diminuata* is potentially intensified via contaminated mechanical vectors (e. g. training targets, hands, clothes of zookeepers) or the environment (e. g. surfaces at resting places), as it has been proposed in former studies (Fravel and Procter, 2016; Pesapane et al., 2018). In zoological gardens, where many different species are held in close proximity, it is highly important, to analyze and evaluate transmission possiblities of pathogens between different species as well as the zoonotic potential. In the last 15 years, no other pinniped species was held at the Vienna Zoo, thereby excluding a potential adequate infestation source for spreading between host species. However, the *O. flavescens* enclosure is located in close proximity to the exhibits of several penguin species, including Humboldt penguins, Northern rockhopper penguins and king penguins. There is a report on a single individual of *H. miroungae* (as *H. erratica* Fain and Mortelmans, 1959) in the larynx of a captive gentoo penguin (*Pygoscelis papua* Forster, 1781), which was held together with Southern elephant seals (Fain and Mortelmans, 1959; Domrow, 1962). Webb et al. (1985) reported on a human case with *O. attenuata*, which was detected under the eye lid of a patient, who was previously sneezed on by a captive walrus during a visit in a marine park. Even though both mentioned cases represent infestations of accidental hosts, accuracy and hygienic measures are important, to minimize the intraspecific transmission risk as well as the zoonotic risk for the zookeepers and veterinarians.

While *O. attenuata* infestations are generally limited to the upper respiratory tract, *O. diminuata* mites colonize the upper (larval/nymphal stages) and the lower respiratory tract (female and male adult mite stages) (Kim et al., 1980). In the present study, all necropsy-detected larval stages were exclusively found in the nasal cavity and in the sinuses. However, during ante mortem conducted BAL, one larval stage was obtained from the lower respiratory tract. Halarachnid mites of pinnipeds are described as viviparous (Furman and Smith, 1973), however, only limited information on the exact reproductive behavior has been published so far. Kim et al. (1980) reported a very small part of the adult *O. diminuata* mite population in the turbinates of Northern fur seals, potentially for reproductive purposes. Hypothetically, the current BAL-findings might indicate that *O. diminuata* larval stages could be born in the depths of the lower respiratory tract, and thereafter transported into sinus systems via nasopharyngeal-directed tracheal ciliary current or active mite movements.

Particularly heavy infestations with high *Orthohalarachne* mite burdens are reported to significantly impact pinniped health and even fatal outcomes are described (Dahme and Popp, 1963; Kim et al., 1980; Petak et al., 2010; Seguel et al., 2018a). High burdens of *O. diminuata* mites in South American fur seals were associated with substantial damage of respiratory tract mucosa and airway obstruction promoting secondary bacterial infections (Seguel et al., 2018a). Additionally, the obligatory migration of nymphal/adult stages to the lower respiratory tract potentially promote bacterial dispersion (Seguel et al., 2018a). Moreover, Dahme and Popp (1963) reported fatal infestations in two captive Californian sea lions caused by *O. diminuat*a (described as *O. letalis* Popp, 1961). Massive aggregations of mites were detected in the entire bronchial tree, associated with exsudative bronchitis and secondary alveolar emphysema and determined as cause of death in both animals (Dahme and Popp, 1963). However, both animals only showed minor symptoms, e.g. reinforced coughing, prior to death (Dahme and Popp, 1963). In the current case, comparable strong pulmonary pathologies were not detected; ante mortem-generated CT-images showed emphysematous bulla-like structures that might indeed be associated with adult *O. diminuata* mites in the lower respiratory tract based on their epithelia-destructive behavior. However, no relevant medical history or any clinically manifested respiratory-associated symptoms were stated for the animal. Nevertheless, in pathology, the nasal mucosa clearly showed hyperemia and multiple petechial hemorrhages, and histology revealed sanioserous exudate and epithelial exfoliation around mite localizations. Further, a low-grade focal *peribronchitis follicularis* was detected, indicating a considerable impairment and destruction of the respiratory system by mite infestation.

Regarding infestation intensities, Kim et al. (1980) reported on very high mite burdens in Northern fur seals revealing mean *Orthohalarachne* spp. numbers of 1808 (ranging from 23 to 8170) in subadult males. In Steller sea lions (*Eumetopias jubatus* Schreber, 1776), Fay and Furman (1982) reported mild inflammation and swelling of tissues caused by *Orthohalarachne* spp. in minor infestations (<20 mites) and significant irritations and damages of parasitized respiratory mucosa in heavier infestations (>20 mites). Referring to this classification system, infestation intensities of *H. halichoeri* in grey seals (*Halichoerus grypus* Fabricius, 1791) stranded along the North Western Spanish Atlantic coast (Alonso-Farré et al., 2012) and of *Orthohalarachne* spp. in stranded South American fur seals in Brazil (Gastal et al., 2016) were evaluated. As such, former authors reported *O. attenuata* in 68.2% of examined South American fur seals, while five individuals (22.7%) showed a co-infestation with *O. diminuata*. Thereby, 13.6% of *O. diminuata* infestations were classified as “mild to moderate” (<20 mites), while 9.1% were recorded as “moderate to severe” infestations (>20 mites) (Gastal et al., 2016). According to this classification scheme (Fay and Furman, 1982), the current infestation has to be classified as severe, since a total of 410 larval/nymphal and 45 adult stages were detected in nasal turbinates and the lower respiratory tract, respectively.

Using their sharp chelicerae and pedipalpes, halarachnid mites can damage respiratory mucosa, thereby potentially generating entry sites for bacteria or viruses promoting secondary infections (Dahme and Popp, 1963, Seguel et al., 2018a). Pesapane et al. (2022) recently characterized the bacteriome of nasopulmonary mites of Southern sea otters and identified a broad spectrum of bacteria, including at least 16 taxa with pathogenic potential. Furthermore, the authors found *Streptococcus phocae* in *Halarachne halichoeri* from Southern sea otters and in *O. attenuata* from California sea lions (Pesapane et al., 2022). Likewise, Seguel et al. (2018a) suspected *O. diminuata* to act as a vector for beta-hemolytic streptococci in South American fur seals. Generally, beta-hemolytic streptococci constitute one of the most frequently isolated bacterial pathogens in marine mammals (Numberger et al., 2021). Interestingly, in the current case, *Streptococcus dysgalactiae equisimilis* was diagnosed in trachea and bronchi by bacteriological analyses. However, up to date*, O. diminuata* has never been examined for its vector role. We thus urge for more research in the field focusing on the pathogenic fauna potentially being transmitted by halarachnid mites.

Up to date, *intra vitam* diagnosis of (ortho-)halarachnosis in captive pinnipeds is challenging. Since most described cases were asymptomatic and reported as incidental findings during necropsy (Vitzthum, 1936; Eichler, 1958; Gretillat, 1959; Dahme and Popp, 1963; Kutzer and Burtscher, 1983), reliable *intra vitam* diagnostic techniques were not developed in the past. However, Fravel and Procter (2016) reported on *O. attenuata* infestations in captive Pacific walruses successfully being diagnosed by voluntary rhinoscopy without the use of sedation, local or general anesthesia. The animals exhibited prominent mucopurulent nasal discharge and were trained for several weeks to accept the nasal insertion of a flexible endoscope. After treatment, this method was also used for therapy control (Fravel and Procter, 2016). In untrained animals, general anesthesia is required to thoroughly inspect the nasal cavity, nasopharyngeal mucosa and lower respiratory tract by endoscopy, however, the risk of general anesthesia cannot be avoided. In the current case, endoscopy of the lower respiratory tract for performing BAL was accessed via the oral cavity. During video-endoscopy, no mites were noticed. However, three *O. diminuata* specimens (two females and one larval stage) were successfully detected during BAL-fluid analysis. Up to date, to the best of our knowledge, there is no established non- or minimally-invasive method for the anesthesia-free detection of this endoparasitosis in Phocidae or Otariidae. Therefore, we call for the development and application of non-invasive sampling methods in free-ranging and captive animals, e. g. by modifying existing techniques for the detection of echinophthiriid lice in pinnipeds (Ebmer et al., 2019) or collection of expectorated mucus.

Although the veterinary importance of nasopulmonary mites has been known for a long time, molecular data are still scarce. Particularly, species of the genus *Orthohalarachne* are molecularly not well characterized. While Pesapane et al. (2018, 2021) provided detailed molecular characterization of *O. attenuata*, *H. halichoeri*, and *H. miroungae*, no molecular data was presently available for *O. diminuata*. Here, we provide the first 16S rRNA gene sequences and we attempted to provide a first preliminary molecular phylogenetical comparison of this species to other Halarachnidae. In addition, we developed an alternative forward primer for the amplification of 16S rDNA sequences of *Orthohalarachne*, since published primers (Black and Piesman, 1994) did not deliver reliable amplification, potentially, due to the heterogeneity at the 5’ end of Halarachnidae 16S rDNA sequences.

Classification and taxonomy of mesostigmatid mites has been a matter of much debate and many revisions. The Halarachnidae are one of 15 families within the superfamily Dermanyssoidea, however many of the included families have not been adequately defined. In a comprehensive phylogenetic study by Dowling and OConnor (2010), one analyzed halarachnid species, *Raillieta caprae* Quintero, Bassols and Acevedo, 1980 clustered with the paraphyletic Laelapidae family in the phylogenetic tree. This clearly highlights that particularly halarachnid phylogeny is not yet fully understood and urges for further studies. We aimed to provide the phylogenetic position of *O. diminuata* within the Halarachnidae family based on the limited available data from GenBank. Our analysis showed monophyly of the *Orthohalarachne* and *Halarachne* genera, however, analysis was only based on one gene region (16S rRNA gene). Considering that the analyzed sequences display a variable part of the 16S rRNA gene and a low number of available sequences were included, phylogenetic results should be interpreted with care. The phylogenetic position within the Halarachnidae and other Dermanyssoidea families should, thus, be addressed in future studies, when more reference sequence data are available and more genes can be inlcuded in the analysis.

## 5 Conclusion

In this study, we here report on a severe *O. diminuata* infestation in a captive subadult South American sea lion, which constitutes the first report of this endoparasitosis in captive *O. flavescens* and one of the few host-parasite records described in general. Since the infested animal as well as all other members of the *O. flavescens* group held at Vienna Zoo were born in captivity, this infestation must have been acquired in the zoo. Even severe infestations may remain asymptomatic for a long period, however, high mite burdens are suspected to impair respiratory mucosa function, enabling inflammation and secondary bacterial, fungal or viral infections of the respiratory tract. We emphasize the high importance of exact and accurate ante and post mortem examinations to detect patent orthohalarachnosis, which most likely represents an underdiagnosed and neglected infestation of pinnipeds in zoological gardens worldwide. Therefore, further research on parasite-driven detrimental effects, the potential vector function, biology and transmission of *O. diminuata* mites is urgently needed to better understand this endoparasitosis in captive pinnipeds. Moreover, the current study urges for establishment and/or modified application of non-invasive diagnostic techniques to detect and adequately sample halarachnid mites of pinnipeds held in zoological gardens to reduce stress and avoid use of anesthesia.

## Ethics approval

The Vienna Zoo approved the analysis of zoo animal population data, pathological results and the publication of this study.

## Funding

Edwin Kniha is a recipient of a DOC Fellowship and is funded by 10.13039/501100001822the Austrian Academy of Science (OeAW).

## Declaration of competing interest

The authors declare that they have no competing interests.
